# Management support for patient safety in two tertiary hospitals in Jamaica: A mixed methods study

**DOI:** 10.1371/journal.pgph.0005332

**Published:** 2025-10-10

**Authors:** Camelia Thompson, Desmalee Holder Nevins, Dawn Walters, Cameal Chin-Bailey, Elon Thompson, Minerva Thame, Kenneth James

**Affiliations:** 1 Department of Community Health and Psychiatry, The University of the West Indies, Mona, Jamaica; 2 Faculty of Medical Sciences, The University of the West Indies, Mona, Jamaica; Islamic Azad University South Tehran Branch, IRAN, ISLAMIC REPUBLIC OF

## Abstract

Management support is important for implementation of policies and procedures, and to create an organizational culture for the delivery of safe patient care. This study sought to determine the culture for management and safety support at two tertiary hospitals in Jamaica, and to explore managers’ involvement in quality and quality improvement activities. A mixed methods study was done among 328 doctors and nurses (quantitative arm) and 17 senior managers (qualitative arm) from two tertiary hospitals. Data on sociodemographic, work-related characteristics and management support for patient safety were collected and in-depth interviews collected explored managers’ involvement in quality and quality improvement activities. The percentage positive score was determined for management support for patient safety and a logistic regression model identified independent predictors of positive scores. Thematic analysis identified themes and subthemes. Overall positive percent score for management support for patient safety was 51.57%. Independent predictors of positive scores were staff position, institution and primary area of work. Doctors were 72.4% less likely than nurses to have positive scores (OR=0.28, 95% CI: 0.14-0.55, p=<0.001). Participants from Institution B were 2.63 times as likely to have positive score compared to participants from Institution A (OR = 2.63, 95% CI: 1.40 – 4.96, p = 0.003). Compared to participants whose primary area of work was medicine, participants from accident and emergency and radiology/laboratory units were 3.15 (95% CI: 1.19-8.35, p = 0.021) and 5.18 (95% CI: 1.82-14.76, p = 0.002) times more likely to have positive scores respectively. Two themes (managers’ role in quality assurance/improvement and challenges in quality assurance and improvement) and six subthemes emerged from in-depth interviews. Institutions should ensure that there is a clear strategy for quality and quality improvement and implement appropriate systems and structures to support quality-related activities. Boards of hospitals should make quality a key item for discussion and action, to ensure good patient outcomes.

## Introduction

Strong management and leadership along with involvement and buy-in from health workers are essential for fostering a culture of patient safety [1]. Managers in health systems have the responsibility for stewardship and governance. Governance refers to the systematic way in which decisions are made and implemented [2], whereas stewardship is the over-arching responsibility of the institutions for the welfare of patients, considering that people entrust their resources and lives to them [3]. Managers are in the position to drive/create policies and procedures that support a quality and safety agenda. They also are influential in creating the organizational culture required for achieving good patient outcomes [4]. While various studies have examined elements of the quality, the governance role in quality has emerged as a topical issue only in recent years.

Clinical governance is the term used to describe the systems put in place by organizations to ensure accountability to communities for continuous improvement in quality of care and for ensuring that care is safe and patient-centered [5]. The concept of clinical governance emerged out of the National Health Service (NHS) United Kingdom (UK), in response to the Bristol heart scandal, where leadership, organizational governance and accountability deficiencies resulted in unnecessary morbidity and mortality among paediatric cardiac surgery patients [6]. The framework of clinical governance from the NHS highlights that the core elements required for achieving quality outcomes are: education and training, clinical audits, clinical effectiveness, research and development, openness and risk management [7,8]. Other frameworks such as the Australian Capital Territory, posit that to achieve person centered, safe and effective care, there needs to be governance, leadership and culture, clinical performance and effectiveness, patient safety and improvement systems, safe environment for care delivery and partnership with consumers. Monitoring, evaluation and improvement should be ongoing [9]. The ultimate aim of clinical governance is quality improvement.

Shaw et al. (2009) examined European hospitals to determine the presence of governance systems and structures. Among the 89 hospitals studied, most hospitals committed to improving their quality in their mission statement. The requirement of unit head reporting on areas of quality was generally low and only 43% of hospitals provided a report on quality to their governing bodies. Most hospitals had a quality committee, but only 66% of them met within the previous year of the study. Many medical staff did not agree that they had ultimate responsibility for the quality of care provided [10].

Studies have explicitly identified the role of the hospital board in quality and quality improvement. Researchers have explored the characteristics of hospital leadership that would most likely strengthen quality improvement activities in hospitals and found that boards that focused more on quality issues had better quality index scores. Better quality index scores were associated with the receipt of formal reports on quality and performance measures, incentives tied to senior executive performance and high interactions with the medical staff on quality improvement performance [11]. Joshi and Hines (2006) looked at hospital board engagement in quality and safety across 30 hospitals in 14 states in the USA and found a positive association between board engagement in quality and good hospital performance. The authors utilized composite measures of rates of pneumonia, heart attack and heart failure to define hospital performance [12].

Jiang et al. (2008), examined how quality practices in board oversight was associated with hospital quality performance. Better performance in mortality and process of care were associated with board practices of: establishment of strategic goals for quality improvement; having a quality committee; having direct involvement in setting the agenda for quality in hospitals; having quality as an agenda item; and linking the performance evaluation of senior executives with select quality indicators [13].

Time spent by boards examining quality influences quality outcome. Joshi and Hines (2006) highlighted inadequate prioritization of quality by hospital boards [14]. Boards typically spend less than half of their time on matters relating to quality [1,11,15–18] and most reported that 25% or less of their time is spent on quality [11,15,17]. Boards that spent greater than 20% of their time discussing quality-related issues had better quality-related outcomes [11,17].

The role of managers in quality has been examined in a systematic review by Parand et al. (2014) [4]. The authors reported that managerial activities relating to quality/patient safety include creating a culture for quality, using data to inform activities and developing strategies specifically aimed at improving quality. Glickman et al. (2007) have reported similar managerial activities being associated with good quality outcomes [19]. In their paper that captured perspectives of managers, key elements critical to promoting quality were: having executive management (senior leadership and board) involvement, development of culture for safety, good organizational design, incentive structures and having the appropriate technology and information management system in place to support quality. Another managerial activity ‘leadership/manager walk-round’ has been highlighted as an approach to improve patient safety culture, as it provides the opportunity to identify and address patient safety issues in a prompt manner [20,21]. Yet literature points to front line/clinical managers in hospital spending limited time on quality [22,23].

This study sought to determine the hospital culture for management and safety support at two tertiary hospitals in Jamaica, and to explore managers’ involvement in quality and quality improvement activities.

## Methods

### Ethics statement

Ethical approvals for the study were received from the Mona Campus Research Ethics Committee, University of the West Indies, Mona (ECP 111, 18/19), and from the South-East Regional Health Authority Ethics Committee, Jamaica. All participants provided written informed consent. The researchers obtained informed consent face-to-face in the presence of an independent witness (not affiliated with the research). The physical form was signed by the researcher, respondent and independent witness.

A mixed-methods study was conducted between 15/03/2019 and 14/06/2019, utilizing a convergent parallel mixed methods design. The quantitative component utilized a cross-sectional study design among 328 doctors and nurses working at two Type A hospitals in the South-East Regional Health Authority in Jamaica. Type A institutions were selected for this study because they serve as the highest-level referral centers for peripheral hospitals and offer the broadest and most comprehensive array of secondary and tertiary healthcare services. Jamaica has three Type A institutions - two located in the South East Regional Health Authority (SERHA) and one in the Western Regional Health Authority (WRHA). However, only the two facilities in SERHA were included in the study, as the WRHA institution was not fully operational during the study period. These institutions typically have a bed capacity between 300 and 500.

The estimated number of doctors and nurses at the institutions studied was 2257. The Agency of Healthcare Research and Quality Hospital Survey on Patient Safety Culture (AHRQ HSOPSC) Report 2018 highlighted a positive percentage score for non-punitive response to errors of 47%; the biggest area for potential improvement of the 12 composites measured among hospitals [24]. Assessment of non-punitive response to errors is a critical component of the determination of patient safety culture, a major objective of this study. Assuming this figure and applying the usual 95% confidence interval, and 5% margin of error, the minimum required sample was 328 [25]. A stratified random sampling method was used to select persons for the sample. Stratification was done by hospitals and by professional groups (doctors and nurses) and the sample was drawn proportionate to size. Where the participant refused to participate in the study, another eligible participant was randomly selected. Twelve potential participants of the 340 approached refused participation in the study and were replaced (refusal rate = 3.5%).

The qualitative component used a phenomenological approach to obtain information from 17 purposively selected senior managers who were working in the position for at least six months at the two institutions. Senior administrators/managers were selected because of their expected knowledge/ability to speak about the systems and structures that were in place to ensure the delivery of quality care to patients. Senior managers have administrative roles and oversight for various categories of workers who are directly or indirectly involved in patient care. The use of 17 participants in the qualitative arm of the study was guided by the principle of data saturation - the point at which no new information is detected. For sample size adequacy, authors recommend the inclusion of between 16 and 24 participants to achieve both code and meaning saturation [26].

For the quantitative component, a self-administered questionnaire was used to capture data on the socio-demographic characteristics (age, gender, and marital status) of health workers, training and work history, staff position, area of work and length of time working in respective positions, hospitals and units among nurses and doctors. The Agency for Healthcare Research and Quality Hospital Survey on Patient Safety Culture (AHRQ HSOPSC) Version 1.0 tool was utilized with permission, for collecting data on the composite ‘management support for patient safety’, which constitutes three items, and scored on a 5-point Likert scale. The composite ‘management support for patient safety’ assesses the extent to which hospital management provides a work climate that promotes patient safety and shows that patient safety is a top priority [27].

A semi-structured interview guide was used by a trained research assistant to direct the qualitative enquiry. Basic demographic and work-related information were obtained from all participants. Six areas were explored which represent characteristics of clinical governance: governance and quality improvement systems; clinical effectiveness; performance and skills management; incident and complaint management; and patient rights and engagement. The questions were guided by the Australian Commission on Safety and Quality in Health Care National Safety and Quality Health Service Standards document [28]. Managers were also asked to highlight their perspectives relating to the challenges in the delivery of quality care. Probing was done as appropriate. All the interviews were audio-recorded, and field notes were made throughout the process. Steps were taken to ensure trustworthiness and the collection of rich, thick data. Such measures included prolonged engagement with participants in the field by interviewing above the data saturation point and allowing enough time for probing and sharing.

Quantitative data were analyzed using the Statistical Package for the Social Sciences (SPSS) Version 21. Descriptive statistics were used to describe participants’ profile and reporting the frequency of the individual items for the ‘management support for patient safety’ composite. A median ‘management support for patient safety’ score was computed and differences in mean by specific variables were examined using Mann Whitney U and Kruskal Wallis tests as appropriate. A logistic regression model was developed to identify independent predictors (sociodemographic and work-related characteristics) of positive management support for patient safety score. For purposes of logistic regression, management support for patient safety scores were reduced to a binary outcome. Composite scores of four to five were coded as positive scores and scores of three and less were coded as negative scores. This dichotomization is commonly applied in patient safety culture research to create meaningful thresholds that align with established benchmarks [29], simplify model interpretation, and ensure adequate sample sizes in each comparison group.

Variables entered in the logistic regression model were those with p-value of 0.10 or less in bivariate analyses. The Hosmer and Lemeshow Test was used to assess goodness of fit. Multicollinearity was evaluated before including variables in the regression models. In cases where multicollinearity was detected, only the variable with the strongest correlation to the outcome (percent positive score) was retained, while the less correlated variable was excluded from the final model. A p-value of 0.05 or less or 95% confidence interval (95% CI) that does not include one in the case of odds ratio, was used to determine independent predictors of positive management support for patient safety score.

For the qualitative component, recorded interviews were transcribed verbatim then analyzed manually for emergent categories consistent with clinical governance themes. A hybrid coding approach was used, combining inductive coding (to allow for new insights to emerge from the data) with deductive analysis guided by the core domains of clinical governance. The process started with the researcher and one additional person on the research team working independently to read and highlight key words and phrases then categorizing and coding these based on the overall clinical governance themes; this was done for the first set of five interview scripts. These initial codes were categorized and compared, and following discussion, a coding framework was agreed upon for use with the remainder of the data set. While this framework was used to ensure consistency, the researchers remained open to new codes or categories that emerged as analysis progressed. Themes were developed by grouping related codes, identifying patterns across transcripts, and considering their relevance to clinical governance constructs. On completion of the process, the findings were summarized, and member checking was done with some participants in the study to ensure that the summaries prepared were an accurate representation of the information they shared during the interview. The preliminary findings were also shared with other research participants/peers for corroboration purposes. Data saturation was reached after ten interviews, at which point no new codes or themes emerged from the data.

Qualitative data were reported thematically. For purposes of confidentiality, only institution name was used when reporting direct quotations. This was done to maintain anonymity of participants as the positions/participants are unique and otherwise may be easily traced. With regard to mixing of data, where qualitative issues address key variables in the quantitative component of the study, both quantitative and qualitative data were matched in joint displays to compare divergence or convergence of the results. Only findings relevant to management support for patient safety are presented in this paper. These finding emerged from the exploration of the clinical governance section related to governance and quality improvement systems. Both quantitative and qualitative data are discussed together.

### Positionality

The research assistant previously worked as a registered nurse at one of the hospitals included in this study, with formal employment ending ten years prior to start of the study. Experience in both the private health sector and academia has contributed to a strong understanding of healthcare systems and service delivery in Jamaica. To minimize potential bias arising from prior professional experience, steps were taken to ensure the integrity of data collection and analysis. Emic perspectives from hospital managers were prioritized through open-ended, semi-structured interviews. Probing questions were used to explore key concepts while avoiding leading questions. The process of bracketing was employed throughout data collection and analysis to consciously set aside preconceptions and maintain analytical rigor. Additionally, memo writing was used as a reflective practice during transcript review, assisting in the identification and separation of the research assistant’s interpretations from the empirical data, thereby enhancing the trustworthiness of the findings.

## Results

### Sociodemographic characteristics of participants

As seen in Table 1, the majority (62.2%) of participants was from Institution B. Most (81.4%) participants were females and 40.2% of participants were in union (married/common-law). The age of participants ranged from 20 years to 64 years with a median age of 31 years (interquartile range [IQR] = 11). Registered nurses constituted the majority (53.7%) of participants. Cumulatively, 39.8% of participants had additional training. Approximately 44% of participants have reported working in their profession from one to five years, and 51.8% reported working in the hospital for a similar period. The largest proportion of participants indicated that their primary area of work was surgery (26.5%) and medicine (22.3%). ‘Other’ included participants from radiology departments and laboratories.

**Table 1:** Socio-demographic Characteristics of Study Participants.

Variables	Frequency % (n)
Institution (n = 328)	
A	37.8 (124)
B	62.2 (204)
Gender (n = 328)	
Male	18.6 (61)
Female	81.4 (267)
Marital Status (n = 328)	
In Union (married/common-law)	40.2 (132)
Not in Union	59.8 (196)
Age Category (n = 305)	
≤ 25 years	15.7 (48)
26-35 years	51.8 (158)
36-45 years	23.6 (72)
≥ 46 years	8.9 (27)
Staff Position (n = 328)	
Registered Nurse	53.7 (176)
Enrolled Assistant Nurse	3.7 (12)
Nursing Sister	5.8 (19)
Medical Intern	3.0 (10)
Senior House Officer	3.4 (11)
Medical Resident	21.3 (70)
Consultant	9.1 (30)
Additional Training (n = 327)	
Post-Basic Nursing	22.3 (73)
Master	2.8 (9)
Doctor of Medicine	10.7 (35)
Other	4.0 (13)
None	60.2 (197)
Years in profession (n = 328)	
<1	5.5 (18)
1-5	43.6 (143)
6-10	24.4 (80)
11-15	13.1 (43)
16-20	6.7 (22)
≥ 21	6.7 (22)
Years in hospital (n = 328)	
<1	8.2 (27)
1-5	51.8 (170)
6-10	18.6 (61)
11-15	10.7 (35)
16-20	5.8 (19)
≥ 21	4.9 (16)
Years in unit (n = 326)	
<1	24.8 (81)
1-5	52.8 (172)
6-10	12.6 (41)
≥ 11	9.8 (32)
Primary area of work (n = 328)	
Medicine (Reference)	22.3 (73)
Surgery	26.5 (87)
Obstetrics & Gynaecology	9.8 (32)
Paediatrics	6.7 (22)
Accident and Emergency	11.3 (37)
Intensive Care Unit/ Operating Theatre	14.3 (47)
Other (Radiology and Laboratory)	9.1 (30)

### Management support for patient safety

Three items were used to examine ‘management support for patient safety’. The overall positive response was 51.57%. Approximately 60% of participants agreed or strongly agreed that the ‘hospital management provides a work climate that promotes patient safety’. Almost 55% were in agreement that the ‘actions of hospital management show that patient safety is top priority’. Only 41% agreed or strongly agreed that their ‘hospital management seem interested in patient safety only after an adverse event happens’ (Table 2).

**Table 2:** Frequency of Responses for Perception on Management Support for Patient Safety.

Management Support for Patient Safety	Frequency % (n)			
	SD/D	N	SA/A	Total
Hospital management provides a work climate that promotes patient safety	20.9 (68)	19.4 (63)	59.7 (194)	100.0 (325)
The actions of hospital management show that patient safety is a top priority	20.6 (67)	24.5 (80)	54.9 (179)	100.0 (326)
Hospital management seems interested in patient safety only after an adverse event happens	40.1 (131)	19.0 (62)	41.0 (134)	100.0 (327)

SD/D= strongly disagree/disagree; SA/A = strongly agree/agree; N=neutral.

### Management support for patient safety score – association with socio-demographic and work-related variables

The composite ‘management support for patient safety’ score was explored to determine whether associations exist with gender, marital status, age category, institution, staff position, primary area of work, length of time in profession, hospital and unit, as well as the number of hours worked per week. Overall, the scores for ‘management support for patient safety’ ranged from one to five with a median score of 3.33 (IQR = 1.33).

There was no statistically significant association between ‘management support for patient safety’ score and marital status (p = 0.076), age category (p = 0.106), length of time in profession (p = 0.084), length of time in unit (p = 0.355) and hours worked per week (p = 0.107). Statistically significant associations were noted between ‘management support for patient safety’ score and gender (U = 6132.0 (z = -3.00), p = 0.003), institution (U = 7619.5 (z = -6.036), p=<0.001), staff category (U = 7284.5 (z = -5.857), p=<0.001), primary area of work (H (6) = 18.66, p = 0.005) and length of time in hospital (H (5) = 13.00, p = 0.023). Management support for patient safety scores were higher for females, institution B, nurses, staff working in ‘other’ areas (radiology and laboratory), and for those working in their hospital for less than one year.

### Independent predictors of positive management support for safety scores

A logistic regression model was developed to identify independent predictors of positive scores for ‘management support and patient safety’. The variables entered in the model were gender, marital status, institution, staff position, primary area of work and length of time in hospital. Due to multicollinearity issue, length of time in profession was excluded from the model. Independent predictors of positive ‘management support and patient safety’ scores were staff position, institution and primary area of work (Table 3).

**Table 3:** Logistic Regression Model for Positive Management Support for Patient Safety Score.

Variables	B	SE	OR (95% CI)
Gender			
Male (Reference)	–	–	–
Female	0.24	0.41	1.27 (0.57-2.87)
Marital Status			
Not in Union	–	–	–
In Union	-0.06	0.28	0.95 (0.54-1.65)
Staff position			
Nurses (Reference)	–	–	–
Doctor	-1.27	0.35	0.28 (0.14-0.55)*
Institution			
A (Reference)	–	–	–
B	0.97	0.32	2.63 (1.40-4.96)*
Length of time in hospital (year)			
<1 (Reference)	–	–	–
1-5	-0.43	0.48	0.65 (0.26-1.65)
6-10	-0.3	0.56	0.74 (0.25-2.22)
11-15	-0.02	0.59	0.98 (0.12-2.18)
16-20	-0.69	0.75	0.50 (0.31-3.11)
≥ 21	0.92	0.76	2.52 (0.57-11.22)
Primary area of work			
Medicine (Reference)	–	–	–
Surgery	0.52	0.4	1.68 (0.76-3.69)
Obstetrics & Gynaecology	0.61	0.54	1.84 (0.64-5.26)
Paediatrics	0.71	0.56	2.03 (0.68-6.10)
Accident and Emergency	1.15	0.5	3.15 (1.19-8.35)*
Intensive Care Unit/ Operating Theatre	0.26	0.48	1.30 (0.51-3.31)
Other	1.65	0.53	5.18 (1.82-14.76)*

*Denotes statistical significance at alpha 0.05 level.

Hosmer and Lemeshow p-value = 0.986; Nagelkerke R square = 0.226.

Compared to nurses, doctors were 72.4% less likely to have positive ‘management support for patient safety’ scores (OR=0.28, 95% CI: 0.14-0.55, p=<0.001). Participants from Institution B were 2.63 times as likely to have positive ‘management support for patient safety’ score compared to participants from Institution A (OR = 2.63, 95% CI: 1.40 – 4.96, p = 0.003). Compared to participants whose primary area of work was medicine, those working in the accident and emergency units and ‘other units” were 3.15 (95% CI: 1.19-8.35, p=0.021) and 5.18 (95% CI: 1.82-14.76, p=0.002) times more likely to have positive ‘management support for patient safety’ scores respectively (Table 3).

### Qualitative findings

In this component, the majority (64.7%) of interviewees was female. Most (58.8%, n = 10) respondents were in the age category 51 years and greater, 17.6% in the 46–50 years age category and 11.8% in the 30–35 age category. There was an equal proportion (5.9%) of senior managers in the 36–40 years and 41–45 years age categories. Participants were working in their capacities for 6 months to 13 years with a median time of 3 years (IQR = 5.45 years).

The themes and subthemes that emerged from the qualitative enquiry are captured in Table 4.

**Table 4:** Themes and subthemes relating to managers role in quality.

Themes	Sub-themes
Managers Role in Quality Assurance/Improvement	Strategic direction and policy development
	Technical oversight
	Involvement of quality in strategic direction of the institution Monitoring and reporting on quality Patient service management
	Administrative and support services
Challenges in Quality Assurance and Improvement	Management and leadership
	Resource availability

### Role in quality assurance/improvement

The role that managers play in clinical governance was explored. **Strategic direction and policy development** formed part of the role that some managers played in ensuring that quality of care is delivered to patients:

So my role is always to ensure we are able to provide continuous delivery of patient care to our patients and to set the strategic goal with the relevant key performance indicators in helping us to monitor and manage our progress in attaining these goals.

[*Institution B*]

… it is about policy. What are some of the policy concerns? What are the policy directives? If somebody has something to suggest, whether it’s coming from nursing or clinical, those kinds of things are presented for our input.[*Institution B*]

Managers noted their role in providing **technical oversight** to staff who are involved directly or indirectly in providing care for patients. The role of oversight included routine monitoring and evaluation of staff to ensure adherence to required standards and also included risk identification, and quality improvement.

I’m also responsible for monitoring through the various processes of clinical quality assurance, evaluation, collection of some of the data, certainly analysis, and then feedback to the Regional Health Authority and to the Ministry of Health and Wellness.

[*Institution A*]

The **involvement of quality in the strategic direction of the institutions** was explored among the managers. It was highlighted throughout, that there is not an overarching strategic plan for the institutions studied. Some participants indicated that they are given targets to meet, but there is no strategic plan.

Well unfortunately, the hospital does not have strategic plan. So, our strategic plan in theory, is embedded in the strategic plan of the Region.*Institution A*]

Ha ha ha! … What strategic plan? Have you seen a strategic plan? So why you asking me questions that nobody knows anything about?[*Institution B*]

I’m not aware of a strategic plan[*Institution B*]

(*whispers*) – we don’t have a strategic plan.[*Institution l A*]

Most participants indicated that they develop department/unit plans to support the delivery of quality care, and some indicated that the lack of an overarching plan presents a challenge.

Of course, that (*lack of a strategic plan*) is a significant gap and lends itself to inefficiencies and challenges with service delivery.[*Institution A*]

So, until we are able to accurately plan for tomorrow, for the amount of patients we intend to see, we are going to continue to have challenges in delivering quality of care.

[*Institution A*]

Participants in the study identified various mechanisms in place for **monitoring and reporting on quality**. These include doing routine walk-throughs, auditing, risk assessment, incident reporting and structured teaching sessions/meetings. Walk-throughs are done in the form of ward rounds, where medical teams discuss patient management and they are able to identify and manage issues relating to quality from that forum. Walk-throughs are also done by the nursing, administrative and support groups to identify day-to-day issues that may impact the delivery of care to patients and appropriate measures are put in place to address the issues identified.

Most participants indicated that they are required to report on elements related to quality and do so in their monthly reports. The reports are sent to heads of department and ultimately to the Chief Executive Officer (CEO). The CEO or technical staff will then determine what is to be reported at the board level.

It was noted from the interviews that at the board level, the focus is more so on ‘serious’ [*high potential for litigation*] matters. There is no specific requirement for reporting on general quality and ‘non-serious’ quality issues at the level of the board. In one institution, managers reported:

No, it’s not a requirement. You normally report on items you consider a significant matter, so right now quality is just mentioned. It’s not one of the core areas we are looking at just now.[*Institution B*]

Quality comes up. We don’t discuss it as a separate entity, but if you take quality to mean, people worrying about waiting time, overnighting in A&E, they are discussed as part of the clinical directors’ report and the nursing report.[*Institution B*]

Of concern at one institution, was the current absence of a board, with an interviewee quipping “the hospital at present doesn’t have a hospital management committee [*board*]”.

**Patient service management** was a key role highlighted in the provision of quality care to patients and involved the management of patient records, scheduling of appointments and registration of birth and deaths. Management of patient complaints, customer service and patient satisfaction evaluation were also roles that were highlighted. The employment and deployment of staff as well as staff welfare were highlighted among the **administrative and support services roles** in quality. Other services such as procurement, janitorial, porter and security services also support the delivery of quality care.

### Challenges with quality assurance and improvement

Participants identified aspects of **management and leadership** that present challenges to quality assurance/improvement. Effective planning and having a strategic focus were identified as two areas to be strengthened.

I want to say that we have to be more strategic. That’s one of the things I would want to put out there, that we need to be strategic in our focus - in our outlook, because some of the things are just ad hoc. I want to see us having more of that type of discussion.

[*Institution A*]

It was also highlighted that executing leadership and administrative functions was a challenge for many managers.

I think the managers will need to... manage and lead our respective areas. We cannot expect other persons to do it for us.[*Institution A*]

Mmmm, one of my pet peeves is to try and get people - people who are promoted into management and supervisory positions to have some pre training for management. You find that some of the doctors, when they get to be the head or in management, they are still doing doctoring and don’t budget enough time to do management of the staff and procedures.

[*Institution B*]

Concern was expressed about the **lack of accountability** within institutions.

… establishing it [*accountability framework*] is difficult because of the culture of the organization and the people. It’s difficult.[*Institution A*]

We have departments where the whole of their equipment is not working… for example, we are trying to find out who is responsible for the morgue. Nobody is responsible for the morgue. They have 4 or 5 refrigeration units and they are all dead [*not operational*].

[*Institution B*]

I believe that persons gravitate to the path of least resistance and so, maybe it is easier to deal with a clerical officer than a consultant. And so, where a clerical officer might misfile a record, you are going to come down and write them up, but whereas somebody does something gravely wrong as it relates to damage to an individual (*patient*), nobody says anything. So that sort of anomaly.[*Institution B*]

Most respondents identified the **lack of resources** as a major challenge to the delivery of quality care. Financial resources were key among the resource challenges which hinder the procurement of supplies and purchase of equipment for care delivery. Competing priorities and ‘misplaced priorities’ were highlighted as two factors hindering the availability of funds to deliver the care required.

Getting things in a timely manner… I mean … you know … the SERHA is the largest region in Jamaica, so you find that everybody sends there, so you find it takes quite a while to get stuff.[*Institution A*]

One of the challenges is that sometimes we do not get all the equipment or supplies we need readily. That can be a challenge at times.[*Institution A*]

Challenges with infrastructure were also reported.

You know physically, we don’t have areas for persons to work from, just now. So physical space … I don’t think we are at the stage where people could work from home to get this thing done successfully.[*Institution A*]

We have challenges too in terms of the design of our building [*hospital*]. It is hot. We push... we move our overcrowding on to the wards. The wards are overcrowded, and it impacts infection control… everybody is miserable in the heat. The beds are old. The rails are not working. The patients are going to fall. [*Institution A*]

## Discussion

Appropriate systems and structures are required to ensure that safe care is delivered to patients. Hospital managers are in a position to develop and implement policies and procedures for patient safety, as well as to create the organizational culture for the delivery of safe care to patients [4]. The overall positive percentage score for management support for patient safety was approximately 52%. This score was 20% lower than the average score in the AHRQ’s 2018 report which covered 630 hospitals. Lower scores have been reported in Portugal [30], Scotland [31] Egypt [32] and Italy [33]. Management support is critical for patient safety because it establishes an organizational culture that prioritizes safety, allocates necessary resources, and drives the implementation of policies and protocols. Strong management engagement also promotes open communication, continuous learning, and staff adherence to safety practices, ultimately improving patient outcomes and reducing errors [19,21]. The study’s finding of a mediocre culture of management support for patient safety reflects a challenge faced by health systems globally. The results reinforce global evidence that management support, through clear strategies, board accountability, and visible leadership is essential for embedding safety as an organizational priority and advancing the WHO Global Patient Safety Action Plan 2021–2030 [34].

The mediocre positive percentage score for management support for patient safety of 52% could be attributed to the lack of a clear strategy for patient safety in both institutions. Senior managers from both institutions reported that they did not have a strategic plan. Strategic plans are critical to provide direction for the organization and to guide the development of approaches to achieve desired goals. The mission statement of both institutions studied highlights their commitment to the delivery of quality care to patients. Given such commitment, a clear strategy addressing quality is required. It was also noted that a Board was not in place at Institution A at the time of the study. At institution B, though the Board was operational, issues relating to quality are reportedly ‘just mentioned’ in board meetings. This area warrants attention to ensure that focus is given to the delivery of quality/safe care in the institutions. There is evidence that board involvement and time spent discussing issues of quality are directly linked to quality patient outcome [4,12,13]. Parand et al. (2014) in a systematic review, highlighted that managers should seek to define strategy, foster culture and use data to advance the quality/safety agenda in institutions [4]. In the qualitative aspect of this study, there were reports of deficits in these areas.

The mission of the Jamaican Ministry of Health and Wellness (MOHW) is “to ensure the provision of quality health service and to promote healthy lifestyle and environmental practices.” Strategic Goal 1 is focused on quality - “safeguarding access to equitable, comprehensive and quality health care” [35]. Yet, there is no clear strategy at the level of the MOHW to guide other institutions. The Pan-American Health Organization (2019) published the “Strategy and plan of action to improve quality of care in health service delivery 2020-2025.” Member States (Jamaica included) are encouraged to: implement continuous processes to improve the quality of care to people, families, and communities in the delivery of comprehensive health services; strengthen the stewardship and governance of health systems to develop a culture of quality; promote sustained quality improvement in the delivery of comprehensive health services; and establish financing strategies that enhance quality of care in the delivery of comprehensive health services [36].

Independent predictors of a positive management support for patient safety score were staff position, institution and primary area of work. Doctors were 72% less likely to have positive ‘management support for patient safety’ scores compared to nurses. At both institutions studied, it was revealed that the quality-related systems were far more established for nurses, and this could be a factor driving their perception of management support. Primary areas of work also emerged as a significant predictor of management support for patient safety. Compared to those working on medicine wards, those working in the accident and emergency department and ‘other’ areas were three and five times more likely to have positive management support for patient safety scores, respectively. High management support for patient safety has been reported in a study of an emergency department in Switzerland [37]. ‘Other’ areas include the laboratory and radiological departments. Given the specialized/technical nature of these areas, managers would be required to have clear standards to support care delivery and adhere to safety/quality standards for routine operation and accreditation. Given the findings in this study, there are opportunities for cross-departmental learning, where structured environments with standardized workflows and accreditation processes may serve as models for other units. Variation across departments also underscores the importance of context-sensitive management/leadership. It has been noted that disciplines such as medicine and the operations of associated wards, may encounter challenges with the application of stricter, less flexible standardized processes due to factors related to patient load, case complexity and staffing shortages [38–40]. These will influence management support for patient safety culture. Context should not be ignored.

Another predictor for positive management support for patient safety score was institution worked; Institution B being 2.63 times more likely than Institution A to have positive scores. Anecdotally, Institution A has in recent times experienced turn-over of senior managers. With high turn-over, there is little continuity, limited ownership and delays in the implementation of existing plans. These factors may impact the staff perception of management support for activities to support the delivery of safe patient care. The management structure at Institution B has been relatively stable, and various risk-related strategies have been implemented in the short term, such as the hiring of dedicated personnel for risk management and quality improvement. Evidence indicates that effective management, characterized by clear delegation of authority and demonstrated competence in coordinating patient safety initiatives, is essential for establishing a robust safety culture, enhancing staff engagement, and promoting compliance with established policies and protocols [41]. Consequently, the observed differences between the two institutions highlight the critical influence of management stability, strategic governance, and sustained operational oversight on staff perceptions and the overall advancement of patient safety.

## Strengths and limitations

This study utilized a mixed-methods approach to understand the culture for management support of patient safety among nurses and doctors, while exploring the systems and structures that are in place for quality/patient safety. By combining quantitative and qualitative data, this approach allowed for a more holistic understanding of the complex factors influencing management support for safety culture, capturing both measurable scores and deeper contextual insights. This methodological triangulation enhanced the validity and richness of the findings, representing a key strength of the study. The sample size was also large. Findings enlarge the limited body of literature and evidence related to culture for management support for patient safety, especially from developing country settings. Only doctors, nurses and senior managers were included in the study from two institutions, limiting generalizability of the finding to hospital workers and other hospitals; the perspectives of other categories of workers and from other hospitals may be different.

## Implication for study findings

This study has important implications for hospital management, policy development, and quality improvement efforts in Jamaica and other low- and middle-income countries, that face similar issues relating to management support for patient safety. The overall mediocre culture of management support for patient safety signals an urgent need for systemic and organizational reform. One key gap identified was the absence of formal strategic planning for quality and safety in both hospitals, which undermines coordinated action and erodes staff confidence in leadership. Hospital boards and senior executives must therefore take the lead in developing clear strategies with defined goals, policy direction, and accountability mechanisms. Prioritizing these actions is essential, as strategic planning provides a roadmap for aligning resources, setting measurable objectives, and fostering a culture of accountability that can drive sustained improvements in patient safety.

The observed differences in perceptions between doctors and nurses suggest limited physician engagement in patient safety initiatives. Strengthening physician involvement through leadership walk rounds, interdisciplinary safety committees, and continuous education in clinical governance may help bridge this gap and embed safety as a shared priority [4,19].

Staff working in radiology and laboratory departments reported stronger perceptions of management support for patient safety. These findings suggest valuable opportunities for cross-departmental learning, with departments that operate using standardized workflows and accreditation protocols, offering potential models for broader organizational improvement. The differences in perceived management support for safety across departments and institutions further emphasize the need for leadership approaches that are responsive to the specific context, culture, and operational realities of each unit.

Although this study focused on two institutions, there is need for policy considerations at the national level. The lack of alignment on hospital quality and safety efforts, suggests the absence of a coordinated national approach. A comprehensive national policy is therefore recommended to articulate clear expectations for governance, strategic planning, and institutional accountability. Such framework would provide a unified framework to guide safety improvements across the health system.

## Conclusion

The culture of management support for patient safety was found to be mediocre, indicating the need for systemic and organizational improvements. While the findings provide useful insights, they should be interpreted with caution, as the study was limited to two referral hospitals and therefore cannot be generalized to all institutions nationally. Strengthening management support for patient safety culture, especially among doctors in leadership roles, requires fostering a deeper appreciation of their responsibility to support safe care delivery. Hospitals must develop strategic plans for quality and safety, backed by effective systems and structures to enable continuous improvement. Specifically, each hospital should establish an active quality and safety committee with clearly defined roles, responsibilities, and regular meetings to monitor performance, address gaps, and implement corrective actions. Leadership at the board and executive levels should prioritize quality and safety as core governance issues, ensuring accountability and coordinated action. Differences in perceptions between professional groups and departments highlight the need for tailored, context-sensitive leadership approaches. Moreover, the stronger safety culture reported in departments with structured workflows suggests valuable opportunities for cross-departmental learning. Beyond the institutions studied, developing a comprehensive national policy that mandates governance structures such as hospital quality committees, reporting mechanisms, and performance indicators is essential to guide and sustain quality and safety improvements across the health system.
